# Administration of *Saccharomyces boulardii* mafic-1701 improves feed conversion ratio, promotes antioxidant capacity, alleviates intestinal inflammation and modulates gut microbiota in weaned piglets

**DOI:** 10.1186/s40104-020-00516-4

**Published:** 2020-12-04

**Authors:** Wenxiu Zhang, Chengling Bao, Jian Wang, Jianjun Zang, Yunhe Cao

**Affiliations:** State Key Laboratory of Animal Nutrition, College of Animal Science and Technology, China Agricultural University, Beijing, 100193 China

**Keywords:** Inflammation, Microbiota, *Saccharomyces boulardii*, Short chain fatty acid, Weaned piglet

## Abstract

**Background:**

Probiotics are used as a means to improve animal health and intestinal development. *Saccharomyces boulardii* is a well-known probiotic; however, few studies have examined the effects of *S. boulardii* on weaned piglet performance. Therefore, this 28-day study compared the effects of *S. boulardii* mafic-1701 and aureomycin in diets for weaned piglets on growth performance, antioxidant parameters, inflammation and intestinal microbiota. One hundred and eight piglets, weaned at 28 d of age (8.5 ± 1.1 kg), were randomly divided into the three dietary treatment groups with six pens and six piglets per pen (half male and half female). The dietary treatment groups were as follows: 1) basal diet (CON); 2) basal diet supplemented with 75 mg/kg aureomycin (ANT); 3) basal diet supplemented with 1 × 10^8^ CFU/kg *S. boulardii* mafic-1701 (SB).

**Results:**

Compared to CON group, SB group had higher feed efficiency (*P* < 0.05) in the last 14 d and lower diarrhea rate (*P* <  0.05) over the entire 28 d. Total superoxide dismutase in serum was markedly increased in SB group (*P* < 0.05). Moreover, compared with CON group, SB group decreased the levels of pro-inflammatory cytokines interleukin-6 (*P* <  0.01) and Tumor necrosis factor-α (*P* < 0.05) in jejunum. Supplementation of *S. boulardii* mafic-1701 increased the abundance of *Ruminococcaceae_UCG_009* and *Turicibacter* (*P* < 0.05), whereas the abundance of unclassified*_Clostridiaceae_4* was decreased (*P* < 0.05). Furthermore, *S. boulardii* mafic-1701 administration increased cecal concentration of microbial metabolites, isobutyrate and valerate (*P* < 0.05).

**Conclusions:**

The improvement in feed conversion ratio, reduction in diarrhea rate in weaned piglets provided diets supplemented with *S. boulardii* mafic-1701 may be associated with enhanced antioxidant activity, anti-inflammatory responses and improved intestinal microbial ecology.

## Background

In order to market pigs sooner and to improve sows’ reproductive performance, the early weaning strategy has been applied in commercial pig production [1]. Weaning is the most stressful period in pig’s life [1]. Some non-antibiotic solutions, including antimicrobial peptides, prebiotics, anti-virulence molecules, antibodies and probiotics, have been developed to maintain the health status of newly weaned piglets [2–4].

Probiotics are defined as “friendly” live microorganisms. When administered in adequate amounts, probiotics can confer a health benefit to the host [5]. *Saccharomyces boulardii* is a safe, efficacious and non-pathogenic yeast isolated from lychee fruit in Indochina; *S. boulardii* belongs to *Saccharomyces cerevisiae* species [6]. However, *S. boulardii* possesses a superior probiotic efficiency than other stains of *Saccharomyces cerevisiae* by exhibiting several distinct physiological and metabolic characteristics [6]. In particular, characteristics of *S. boulardii* that make it suitable for use in weaned piglet diets include heat tolerance and resistance to gastric acidity, bile and proteolysis [7, 8]. The degree of acid tolerance and resistance to enzyme digestion suggest that *S. boulardii* may be suited for survival in the intestines. In addition, accumulating evidence suggests oral administration *S. boulardii* may protect animals against antibiotic-associated diarrhea and *Clostridium difficile*-associated colitis in animal models [9, 10]. In human studies, administration of *S. boulardii* protected humans against *Clostridium difficile* infection, mitigated intestinal microbiota disorder and reduced antibiotic-associated diarrhea [11, 12].

The beneficial properties mentioned here indicate that *S. boulardii* would be used as a promising probiotic-based feed additive in animal production. However, the effects of *S. boulardii* on weaned piglets remain unclear. Therefore, the objective of this study was to determine whether *S. boulardii* mafic-1701 supplementation to weaned piglet diets would improve feed conversion ratio, antioxidant capacity in serum, gut anti-inflammatory responses, microbiota composition and fermentation metabolites concentrations in weaned piglets.

## Materials and methods

Experimental protocols of animal handling and dietary treatments were approved by the “Institutional Animal Care and Use Committee of China Agricultural University” (ICS 65.020.30). All animal procedures were carried out in accordance with the specifications of the National Research Council’s Guide for the Welfare and Ethics of Laboratory Animals.

### Probiotic strain and culture conditions

The yeast *S. boulardii* mafic-1701 was isolated by our laboratory and maintained on yeast extract peptone dextrose agar plates to screen single colonies. Colonies of *S. boulardi* mafic-1701 were inoculated in yeast extract peptone dextrose medium for 16 h at 37 °C to prepare seed cultures. High density fermentation cultivation was performed using a fermentor (30 L) with an initial volume of 15 L of medium with the following composition (g/L): dextrose, 50; corn steep liquor powder, 25; (NH_4_)_2_SO_4_, 4; KH_2_PO_4_, 2; MgSO_4_, 0.5. 750 mL of seed cultures were added into medium. The initial dissolved oxygen concentration was adjusted to 30%. The pH was set at 6.5 using 3 mol/L NaOH. Fermentation was processed at 37 °C at 250 r/min with an aeration rate of 5 L/min of air. The pH was maintained at 6.5 by the addition of 3 mol/L NaOH and anti-foaming agents were automatically added when each time foam was generated. Samples were collected every 12 h to measure the biomass of *S. boulardii* mafic-1701 fermented. The yeast product used in this present study was obtained by mixing the precipitate of the fermentation broth with 21.57 kg wheat bran [13]. The final product moisture content was controlled at 2% by drying at the temperature of 37 °C.

### Experimental design and diets

The experiment was conducted at Feng Ning Swine Research Unit of China Agriculture University (Academician Workstation in Chengdejiuyun Agricultural & Livestock Co., Ltd). The experiment was conducted as a completely randomized design. A total of 108 piglets (Duroc × Landrace × Yorkshire) were weaned at 28 d of age (8.5 ± 1.1 kg), and randomly assigned to one of three dietary treatment groups, based on their gender and initial body weight. Treatment diets included basal diet (CON), basal diet supplemented with 75 mg/kg aureomycin (Chia Tai Group, Henan, China) (ANT) [14] and basal diet supplemented with 1 × 10^8^ CFU/kg *S. boulardii* mafic-1701 (SB). Basal diets (Table 1) in this study were formulated to meet or exceed NRC (2012) nutritional requirements of piglets in 2 phases (d 0–14 and d 15–28) after weaning. Each treatment group consisted of 6 replicate pens and each pen consisted 3 male and 3 female piglets. All piglets were housed in 1.2 m × 2.1 m pens equipped with plastic leakage dung floors and were allowed *ad libitum* access to water and feed. Room temperature setpoint was 26 °C on the day of weaning and gradually decreased to 22 °C within the first week after weaning. The humidity was held constant at 65–75%.

**Table 1:** Composition and nutrient levels of basal diets (as-fed basis)

	Diet	
Items^a^	d 0–14	d 15–28
Ingredient, %		
Corn	59.82	64.32
Soybean meal	15.00	15.80
Extruded soybean	6.30	6.00
Fish meal	4.00	3.50
Whey powder	4.00	3.15
Soybean protein concentrate	4.80	2.80
Soybean oil	2.20	0.90
Dicalcium phosphate	1.15	1.00
Limestone	0.82	0.60
Salt	0.30	0.30
*L*-lysine HCl	0.52	0.44
Methionine	0.18	0.12
Threonine	0.18	0.14
Serine	0.03	0.03
Chromic oxide	0.00	0.03
Choline chloride	0.20	0.10
Vitamin-mineral premix^b^	0.50	0.50
Total	100.00	100.00
Selected nutrient level, calculated^c^		
Digestible energy, Mcal/kg	3.55	3.48
SID^d^ Lysine, %	1.39	1.25
SID Methionine, %	0.49	0.41
SID Threonine, %	0.96	0.74
SID Serine, %	0.26	0.22
Crude protein, %	20.81	19.53
Calcium, %	0.84	0.70
Total phosphorous, %	0.65	0.61

^a^Experimental diets were control diet (CON), CON + 75 mg/kg aureomycin (ANT), CON + 1 × 10^8^ CFU/kg *S. boulardii* mafic-1701 (SB)

^b^The vitamin-mineral premix contained (per kilogram of complete diet): vitamin A, 9000 IU; vitamin D_3_, 3000 IU; vitamin E, 20.0 IU; vitamin K_3_, 3.0 mg; vitamin B_1_, 1.5 mg; vitamin B_2_, 4.0 mg; vitamin B_6_, 3.0 mg; vitamin B_12_, 0.2 mg; niacin, 30.0 mg; pantothenic acid, 15.0 mg; folic acid, 0.75 mg; biotin, 0.1 mg; Fe (FeSO_4_·H_2_O), 75.0 mg; Cu (CuSO_4_·5H_2_O), 150 mg; Zn (ZnSO_4_·7H_2_O), 90 mg; Mn (MnSO_4_), 60.0 mg; I (KI), 0.35 mg; Se (Na_2_SeO_3_), 0.30 mg

^c^Values were calculated according to NRC (2012). ^d^SID: standardized ileal digestible

### Performance and diarrhea incidence

Piglets and feeders were weighted on d 0, 14 and 28. Average daily gain (ADG), average daily feed intake (ADFI) and feed to gain ratio (F:G) were calculated on a pen basis. To evaluate the rate of diarrhea, fecal consistency was visually assessed three times per day throughout the experiment by fixed observers blind to the treatment according to the method described by Hart and Dobb [15]. The scoring system was applied to determine the rate of diarrhea as following: 1 = normal feces; 2 = possible slight diarrhea; 3 = fluid feces; 4 = very watery diarrhea [16]. The occurrence of diarrhea was defined as maintaining fecal scores of 3 or 4 for 2 consecutive days [16]. The rate of diarrhea was calculated according to the following formula: the rate of diarrhea (%) = (number of piglets with diarrhea × diarrhea days)/(number of piglets × total observational days) × 100 [16].

### Sample collection and processing

On the d 28, one piglet from each replicate pen close to the median body weight was selected for sampling. Blood (7 mL) was collected via jugular venipuncture using vacutainer without anticoagulant (Greiner Bio-One GmbH, Kremsmunster, Austria) [17], which was subsequently centrifuged at 3000×*g* for 15 min for serum preparation and stored at − 80 °C until further analysis.

Three piglets per treatment group were randomly selected for slaughter. The selected piglets were from the different pens and their body weights were close to the median body weight [14]. Approximately 10 g digesta from the mid cecum and colon of each piglet were collected in sterile tubes, flash frozen in liquid nitrogen and stored at − 80 °C until further analysis [13]. One aliquot of digesta samples were obtained for microbial composition analysis and additional subsamples were taken to determine the short chain fatty acids (SCFAs) in the gut. Intestinal tissues (3.0 cm) were respectively taken from jejunum and ileum, washed with normal saline to remove gut contents, immediately preserved in liquid nitrogen and kept at − 80 °C for anti-inflammatory analysis.

### Serum immune and antioxidant parameters

Serum immunoglobulins (IgA and IgG) were analyzed using commercially available ELISA kits following manufacturer’s instructions (Nanjing Jiancheng Bioengineering Institute, Nanjing, China). The antioxidant capacity based on serum concentrations of total superoxide dismutase (T-SOD), malondialdehyde (MDA), total antioxidant capacity (T-AOC) and glutathione peroxidase (GSH-Px) were assessed using commercially available ELISA kits according to manufacturer’s instructions (Nanjing Jiancheng Bioengineering Institute, Nanjing, China).

### Cytokine measurement

The tissue concentrations of interleukin-8 (IL-8), interleukin-4 (IL-4), interleukin-6 (IL-6) and tumor necrosis factor-α (TNF-α) were determined with commercially available ELISA kits following the manufacturer’s instructions (Nanjing Jiancheng Bioengineering Institute, Nanjing, China). Briefly, samples of the jejunum and ileum tissues were thawed and homogenized in PBS (1:9 wt/vol, pH 7.4) and centrifuged at 2000×*g* for 20 min. The supernatant was collected for the determination.

### Microbiota analysis

Microbial community genomic DNA was isolated from cecal and colonic digesta, using the E.Z.N.A.® stool DNA kit (Omega Bio-tek, Norcross, GA, USA) according to the manufacturer’s specifications. The V3-V4 regions of the bacterial 16S rRNA gene were amplified by PCR using universal primers 338F (5′-ACTCCTACGGGAGGCAGCAG-3′) and 806R (5′-GGACTACHVGGGTWTCTAAT-3′) with the following procedures: initial denaturation at 95 °C for 3 min, followed by 27 cycles of denaturing at 95 °C for 30 s, annealing at 55 °C for 30 s, extension at 72 °C for 45 s, single extension at 72 °C for 10 min and end at 4 °C [18]. Illumina sequencing was performed, raw data were quality-filtered using Trimmomatic and merged by FLASH software with the following criteria: 1) average quality score less than 20 were truncated. A 50-bp sliding window was set and reads shorter than 50 bp or containing ambiguous reads were discarded; 2) sequences longer than 10 bp were assembled based on their overlapped sequence. The maximum mismatch ratio of overlap area was 0.2. Unassembled reads were discarded; 3) samples were distinguished according to their barcode and primers, and the reads with ambiguous bases were removed [18].

Using UPARSE (version 7.1, http://drive5.com/uparse/) operational taxonomic units (OTUs) with 97% similarity cutoff were clustered and chimeric sequences were filtered out. Each 16S rRNA representative gene sequence was categorized and analyzed by RDP Classifier (http://rdp.cme.msu.edu/) against the Silva (SSU128) 16S rRNA database using confidence threshold of 70% [13].

### Quantification of fermentation products

The concentrations of SCFAs were assayed as literature reported [4, 14]. Briefly, approximately 0.5 g of intestinal digesta was weighed into a 10-mL polypropylene tube and diluted 1:16 with ultrapure water (8 mL). Glass spheres were added and vortexed to homogenize the contents. Polypropylene tubes were paced in an ultrasonic bath (KQ5200DE; Kunshan Ultrasonic Instrument, Jiangsu, China) at room temperature for 30 min. Then, the mixture was centrifuged at 4000×*g* for 15 min. Next, 0.16 mL of supernatant transferred into a 10-mL tube with 7.84 mL ultrapure water and filtered through a 0.22-μm filter. The SCFAs in a 25-μL extracted sample solution were determined by high performance ion chromatography (ICS-3000; Dionex, USA) with a conductivity detector. Finally, the concentrations of SCFAs were calculated and normalized to intestinal digesta weight as milligrams per kilogram.

### Statistical analysis

Replicate (pen) was considered the experimental unit for analysis of differences in growth performance and diarrhea rate. Individual piglets were considered the experimental unit for analyses of serum immunoglobulins, antioxidant parameters, gut inflammatory parameters, microbiota, and SCFAs. Growth performance, serum immune, antioxidant parameters, inflammatory parameters and SCFAs were analyzed by one-way ANOVA using Bonferroni test (SPSS Inc., Chicago, IL, USA). Diarrhea rate were analyzed by Chi-square test (SPSS Inc., Chicago, IL, USA) [19, 20]. The bacterial community at the level of phylum, family and genus were analyzed by Kruskal-Wallis method followed by Welch’s test [13, 20]. Probability values of *P* < 0.05 were considered statistical significance.

## Results

### Growth performance and diarrhea incidence

The effects of dietary treatment on ADFI, ADG and F:G are presented in Table 2. There were no significant differences in ADFI and ADG among three treatment groups (*P* > 0.05). However, compared with CON group, SB group had lower F:G during d 15 to 28 (*P* < 0.05) and d 0 to 28 (*P* < 0.01). The rate of diarrhea was significantly associated with the dietary treatment (Table 3). Over the entire 28 d, SB group markedly decreased the rate of diarrhea compared to CON group (*P* < 0.05).

**Table 2:** Effect of *S. boulardii* mafic-1701 on growth performance in weaned piglets^*^

Items	CON	ANT	SB	SEM	*P*-value
d 0 body weight, kg	8.4	8.6	8.5	0.26	0.97
d 14 body weight, kg	12.8	13.6	13.0	0.38	0.71
d 28 body weight, kg	20.1	22.1	21.4	0.57	0.37
d 0 to 14					
ADG, g/d	311.6	347.4	320.1	10.58	0.38
ADFI, g/d	488.6	527.7	476.9	16.25	0.44
F:G	1.58	1.52	1.47	0.04	0.56
d 15 to 28					
ADG, g/d	511.0	565.2	579.1	17.52	0.28
ADFI, g/d	1096.4	1204.7	1112.5	33.77	0.39
F:G	2.17^a^	2.14^ab^	1.92^b^	0.04	0.02
d 0 to 28					
ADG, g/d	421.7	490.0	463.3	13.99	0.31
ADFI, g/d	810.0	873.3	810.0	22.64	0.42
F:G	1.92^a^	1.82^b^	1.78^b^	0.02	< 0.01

^*^n = 6 per pen, In the same row, experimental diets were control diet (CON), CON + 75 mg/kg aureomycin (ANT), CON + 1 × 10^8^ CFU/kg *S. boulardii* mafic-1701 (SB). In the same row, values with different lowercase letter superscripts mean significant difference (*P* < 0.05)

**Table 3:** Effect of *S. boulardii* mafic-1701 on the rate of diarrhea (%) in weaned piglets^*^

Experiment phases	CON	ANT	SB	*P*-value
d 0 to 14	20.24^a^	11.11^b^	11.31^ab^	0.02
d 15 to 28	7.14^a^	3.17^b^	4.37^a^	0.03
d 0 to 28	13.69^a^	7.14^b^	7.84^b^	< 0.01

^*^n = 6 per pen, experimental diets were control diet (CON), CON + 75 mg/kg aureomycin (ANT), CON + 1 × 10^8^ CFU/kg *S. boulardii* mafic-1701 (SB). The rate of diarrhea was calculated according to the following formula: the rate of diarrhea (%) = (number of piglets with diarrhea × diarrhea days)/(number of piglets × total observational days) × 100. In the same row, values with different lowercase letter superscripts mean significant difference (*P* < 0.05)

### Serum immune and antioxidant parameters

The serum concentration of T-SOD was increased in SB group than that of piglets in CON group (*P* < 0.05) (Table 4). Moreover, the serum concentrations of T-AOC, MDA and GSH-Px did not significantly differ among three treatment groups. There were no significant differences in the serum concentrations of IgA and IgG among three treatment groups (Table 4).

**Table 4:** Effect of *S. boulardii* mafic-1701 on serum immune and antioxidant parameters in weaned piglets^*^

Items	CON	ANT	SB	SEM	*P*-value
IgA, g/L	1.00	1.38	1.30	0.09	0.16
IgG, g/L	10.01	11.24	13.10	1.51	0.72
T-SOD, U/mL	190.10^a^	207.44^ab^	224.59^b^	5.21	0.02
MDA, nmol/mL	2.33	1.74	1.59	0.14	0.07
T-AOC, mmol/L	0.24	0.28	0.30	0.02	0.36
GSH-P_X_, U/mL	634.15	670.15	664.61	12.54	0.48

^*^Experimental diets were control diet (CON), CON + 75 mg/kg aureomycin (ANT), CON + 1 × 10^8^ CFU/kg *S. boulardii* mafic-1701 (SB). Blood was collected from one piglet selected from each replicate. In the same row, values with different lowercase letter superscripts mean significant difference (*P* < 0.05)

### Intestinal inflammatory responses

The levels of TNF-α (*P* < 0.01) and IL-6 (*P* < 0.05) in jejunum were decreased significantly in ANT group compared to CON group (Table 5). Similarly, SB group markedly decreased the levels of TNF-α (*P* < 0.05) and IL-6 (*P* < 0.01) in jejunum. In addition, no significant differences were observed on the levels of IL-8 and IL-4 among three treatment groups (*P* > 0.05).

**Table 5:** Effect of *S. boulardii* mafic-1701 on inflammatory parameters in jejunum and ileum in weaned piglets^*^

Items	CON	ANT	SB	SEM	*P*-value
TNF-α, ng/L					
Jejunum	200.18^a^	112.57^b^	117.33^b^	16.60	< 0.01
Ileum	101.59	123.24	157.42	13.38	0.31
IL-8, ng/L					
Jejunum	133.06	108.04	128.97	5.36	0.07
Ileum	286.76	160.73	177.90	25.12	0.06
IL-6, ng/L					
Jejunum	263.60^a^	143.04^b^	92.64^b^	28.25	< 0.01
Ileum	111.61	111.66	132.87	8.25	0.60
IL-4, ng/L					
Jejunum	88.74	101.35	125.38	7.74	0.14
Ileum	87.31	99.18	105.40	3.64	0.06

^*^Experimental diets were control diet (CON), CON + 75 mg/kg aureomycin (ANT), CON + 1 × 10^8^ CFU/kg *S. boulardii* mafic-1701 (SB). Intestinal tissues were collected from three piglets per treatment. In the same row, values with different lowercase letter superscripts mean significant difference (*P* < 0.05)

### Intestinal microbiota composition

**Fig. 1:**
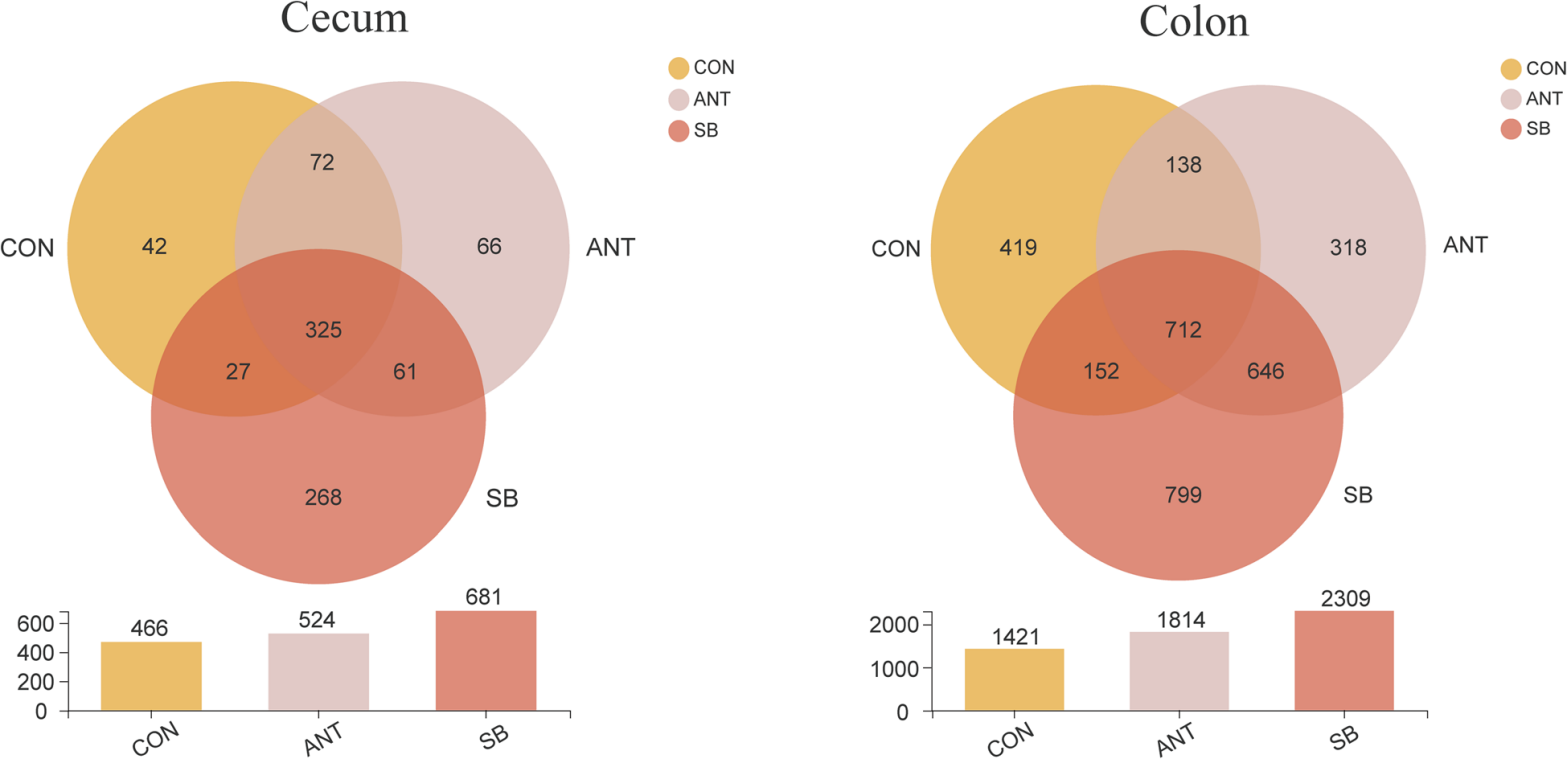
The bacterial operational taxonomic units community composition of the cecum and colon in weaned piglets. Venn diagrams of the bacterial operational taxonomic units community among three treatment groups: control diet (CON), CON + 75 mg/kg aureomycin (ANT), CON + 1 × 10^8^ CFU/kg *S. boulardii* mafic-1701 (SB)

**Fig. 2:**
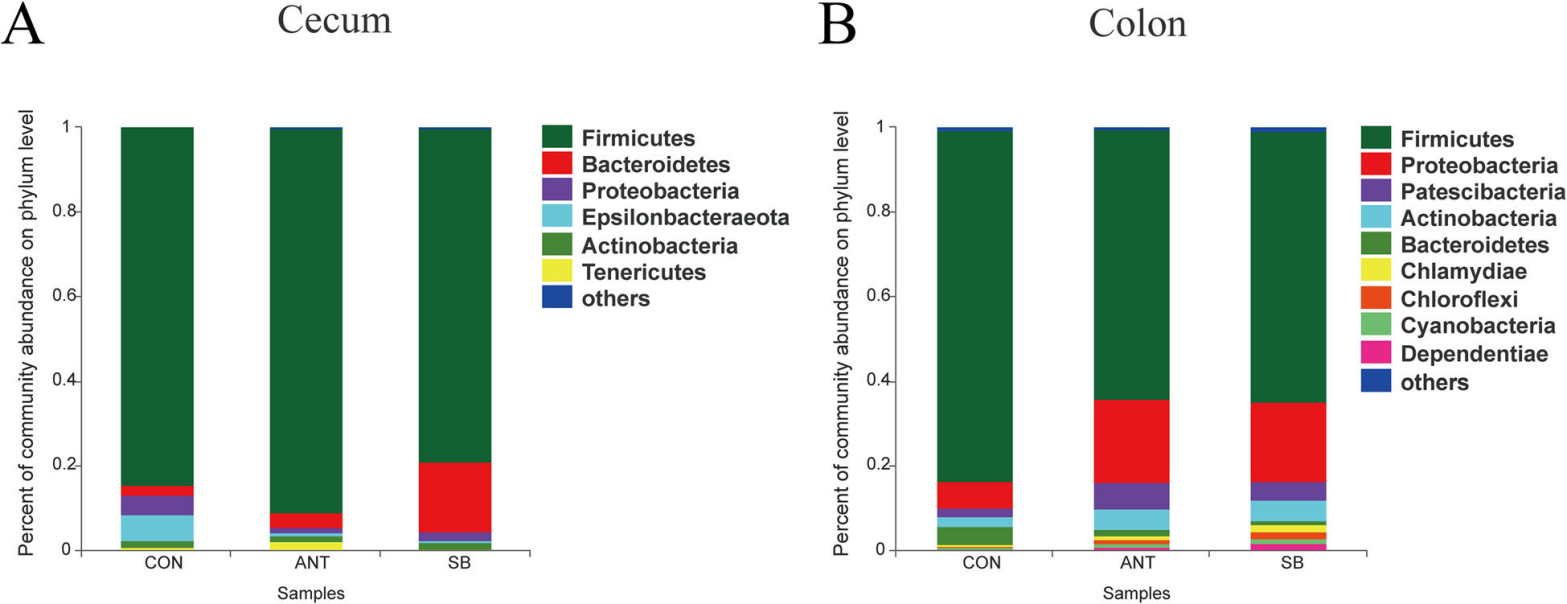
Characterization of communities on phylum level. Experimental diets were control diet (CON), CON + 75 mg/kg aureomycin (ANT), CON + 1 × 10^8^ CFU/kg *S. boulardii* mafic-1701 (SB). Effects of diet treatment on intestinal bacterial communities of weaned piglets at the phylum level. **a** Community barplot analysis for the cecal bacterial communities. **b** Community barplot analysis for the colonic bacterial communities

The OTUs were classified for bacterial community on the basis of usable sequence at 97% similarity. The analysis of OTUs in the cecal and colonic digesta are shown in Fig. 1. There were 42, 66, 268 unique OTUs in the cecal digesta of the CON group, ANT group, and SB group respectively and a total of 325 OTUs were common to all treatment groups. In the colonic digesta, 712 OTUs were common among the three treatment groups with 419, 318, 799 OTUs unique to CON group, ANT group and SB group, respectively. Figure 2 depicts the microbial composition of cecal and colonic digesta across three treatment groups. In the cecal digesta, Firmicutes was the most predominant phylum among the three treatment groups, and Bacteroidetes was the second abundant phylum in ANT group and SB group. Figure 2 also shows that Firmicutes and Proteobacteria were the dominant phyla in the colonic digesta.

**Fig. 3:**
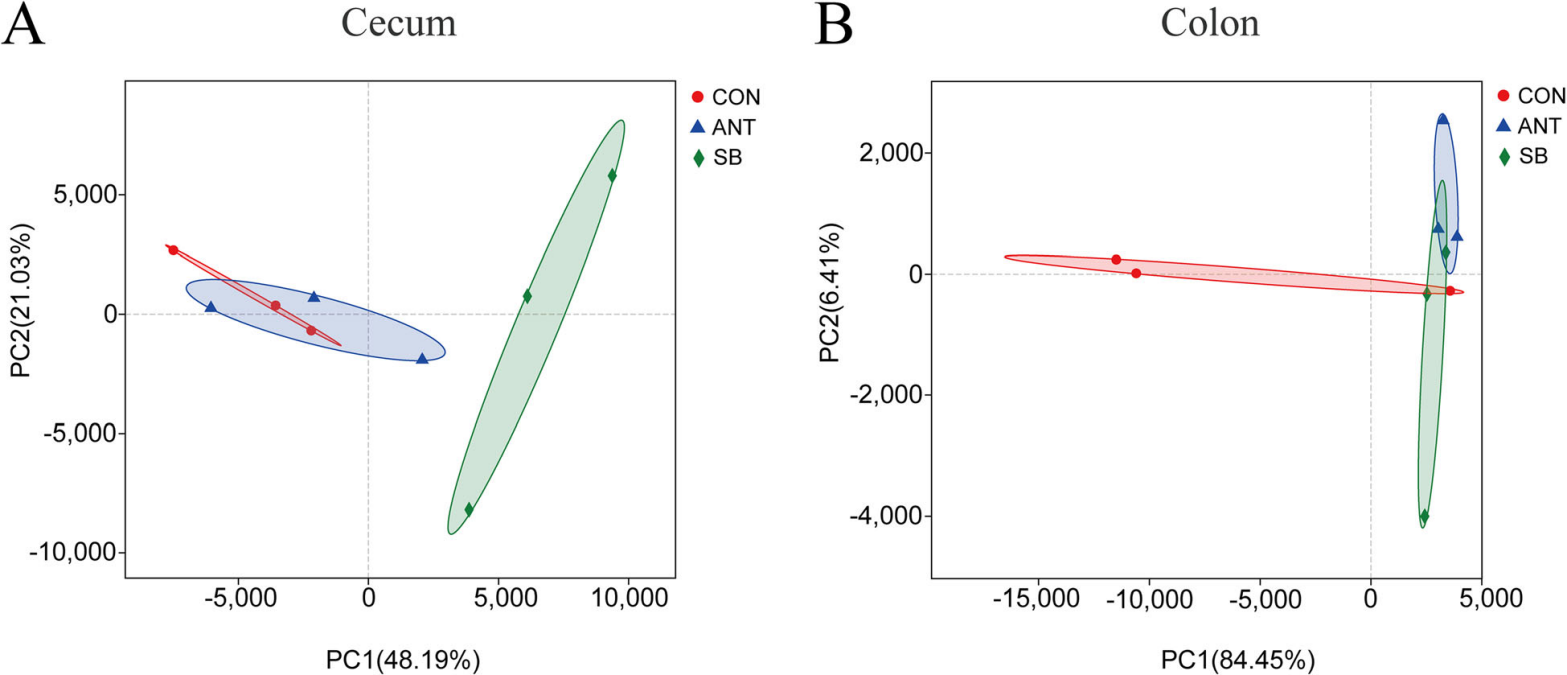
Principal component analysis (PCA) of bacterial community. Experimental diets were control diet (CON), CON + 75 mg/kg aureomycin (ANT), CON + 1 × 10^8^ CFU/kg *S. boulardii* mafic-1701 (SB), Different symbols represent different treatment groups. **a** PCA plot for the cecal bacterial communities. **b** PCA plot for the colonic bacterial communities

Principal component analysis (PCA) based on Bray-Curtis distances indicated that SB group was distinctly separated in comparison to CON group and ANT group in the cecal microbiota (Fig. 3). Whereas, the colonic digesta of SB group were clustered with ANT group, which indicated that the colonic microbiota composition of ANT group and SB group was more similar.

**Fig. 4:**
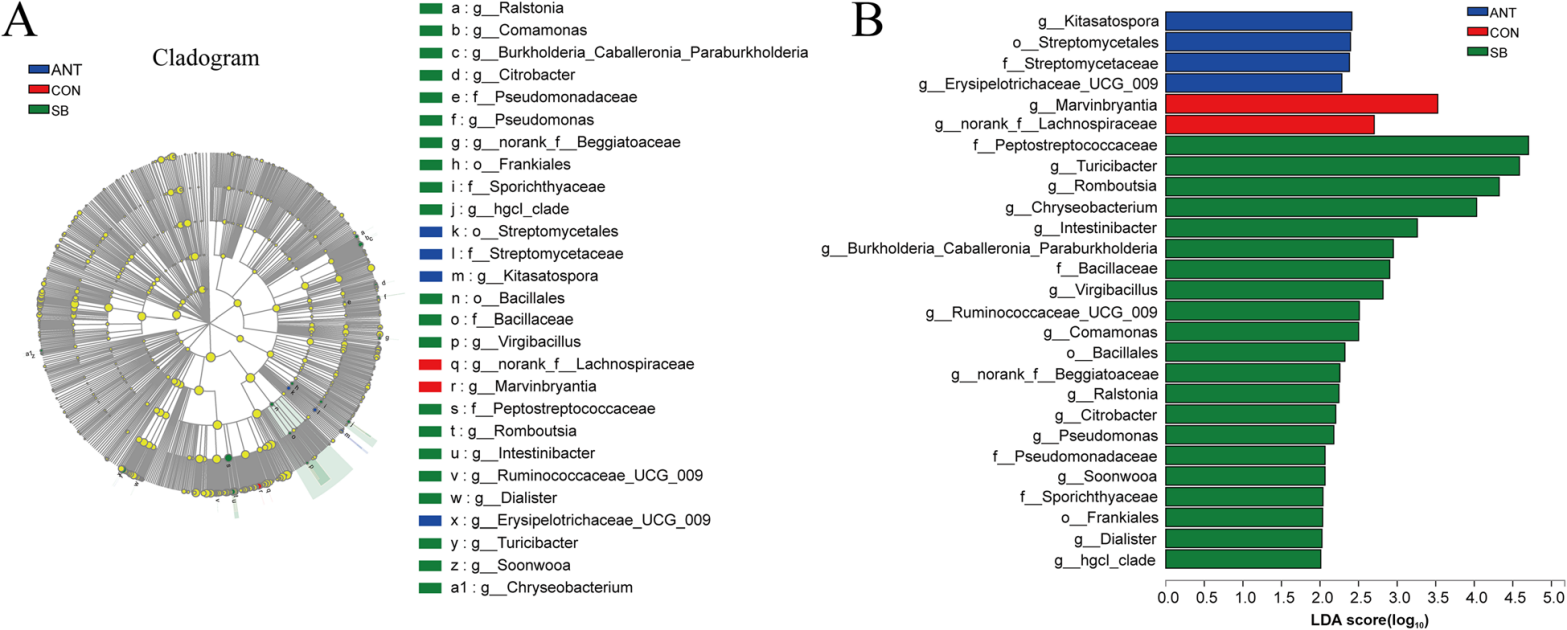
Different structures of cecal bacterial communities from phylum to genus level among three treatment groups*.* Experimental diets were control diet (CON), CON + 75 mg/kg aureomycin (ANT), CON + 1 × 10^8^ CFU/kg *S. boulardii* mafic-1701 (SB). **a** Taxonomic representation of distinct bacterial with statistically significant higher abundances. **b** Histogram of linear discriminant analysis plots indicate scores for differentially abundant genera

**Fig. 5:**
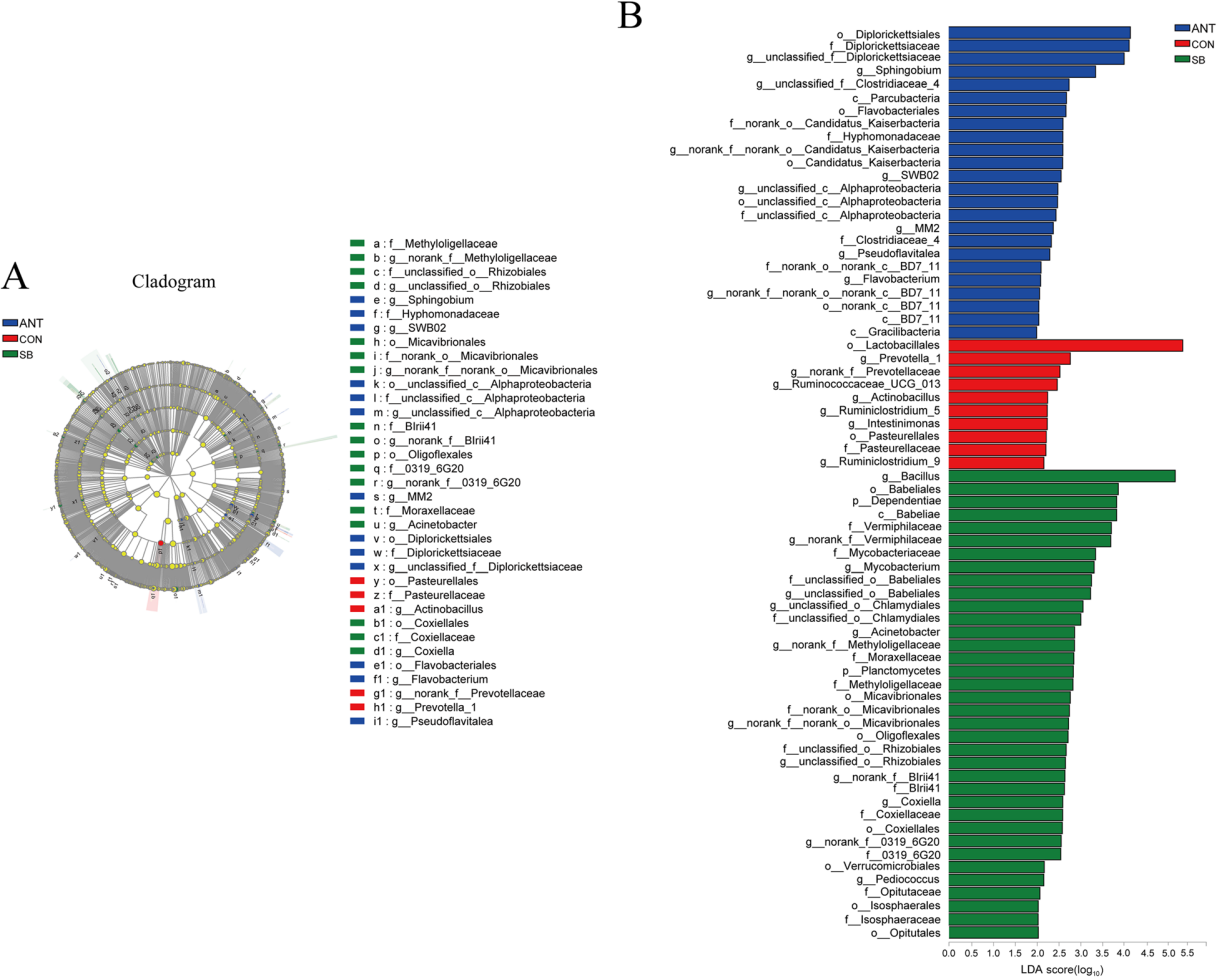
Different structures of colonic bacterial communities from phylum to genus level among three treatment groups*.* Experimental diets were control diet (CON), CON + 75 mg/kg aureomycin (ANT), CON + 1 × 10^8^ CFU/kg *S. boulardii* mafic-1701 (SB). **a** Taxonomic representation of distinct bacterial with statistically significant higher abundances. **b** Histogram of linear discriminant analysis plots indicate scores for differentially abundant genera

Differences in the relative abundance of microbiota in the cecal and colonic digesta among three treatment groups are shown in cladograms, and the linear discriminant analysis (LDA) scores of 2.0 or higher were confirmed by the linear discriminant analysis effect size (LEfSe). In the cecal digesta (Fig. 4), the abundance of Bacillaceae family and Bacillales order were significantly increased in SB group (*P* < 0.05). Moreover, *Ruminococcaceae_UCG_009* and *Turicibacter* genus were enriched in SB group (*P* < 0.05). In the colonic digesta (Fig. 5), the proportion of *Bacillus* genus was significantly increased in SB group (*P* < 0.05), while greater relative abundance of Lactobacillales order and *Prevotella*_*1* genus were observed in CON group (*P* < 0.05) In addition, the abundance of unclassified*_Clostridiaceae_4* genus and its family Clostridiaceae_4 were significantly enriched in ANT group (*P* < 0.05).

### Concentrations of fermentation metabolites

SCFAs in the cecal and colonic digesta are presented in Table 6. The results showed that SB group had higher concentrations of isobutyrate and valerate in the cecal digesta than piglets in CON group (*P* < 0.05).

**Table 6:** Effect of *S. boulardii* mafic-1701 on the concentrations of SCFAs (mg/kg) in weaned piglets^*^

Items	CON	ANT	SB	SEM	*P*-value
Acetate					
Cecum	3724.49	4052.51	3494.39	112.17	0.13
Colon	4102.98	4527.22	4245.64	74.78	0.05
Propionate					
Cecum	2241.26	2484.82	2812.71	221.88	0.60
Colon	2686.08^a^	3252.93^b^	2861.84^a^	81.83	< 0.01
Formate					
Cecum	36.77	62.54	72.37	6.43	0.06
Colon	58.27	63.13	68.00	4.20	0.67
Isobutyrate					
Cecum	2.89^a^	5.10^ab^	15.60^b^	2.42	0.03
Colon	42.35	44.60	28.09	3.26	0.05
Butyrate					
Cecum	870.88	1114.59	1204.74	87.96	0.29
Colon	1499.63^a^	2105.30^b^	1833.76^ab^	97.96	0.03
Isovalerate					
Cecum	2.15^a^	11.67^b^	5.47^ab^	1.64	0.04
Colon	36.80	36.70	22.72	4.55	0.35
Valerate					
Cecum	92.16^a^	211.31^b^	235.98^b^	24.69	0.01
Colon	250.90^a^	460.89^b^	258.83^a^	35.87	0.01

^*^Experimental diets were control diet (CON), CON + 75 mg/kg aureomycin (ANT), CON + 1 × 10^8^ CFU/kg *S. boulardii* mafic-1701 (SB). Cecal and colonic digesta were collected from three piglets per treatment and the concentrations of SCFAs were measured. In the same row, values with different lowercase letter superscripts mean significant difference (*P* < 0.05)

## Discussion

*S. boulardii* is an important species of microorganism, which has known positive effects on gut health of human [11, 12]. Unfortunately, data on the effect of *S. boulardii* on weaned piglets are limited. Therefore, in this study we investigated the effects of *S. boulardii* mafic-1701 supplemented in the diet on weaned piglet health and gut microbiota composition over 4 weeks. The dose of *S. boulardii* mafic-1701 was selected according to the studies by Kamm et al. [21] and Hancox et al. [22] The two doses of *S. boulardii* in their studies were 1 × 10^7^ CFU/kg and 1 × 10^9^ CFU/kg, respectively. We selected 1 × 10^8^ CFU/kg, the middle dose of *S. boulardii* of the two studies as the experimental treatment in this study.

In the present study, supplementation of *S. boulardii* mafic-1701 improved feed conversion ratio compared with CON group. A previous study reported that administration of yeast improved feed conversion ratio of weaned piglets [23], which is in agreement with our results. Reports on the effect of dietary supplementation of *S. boulardii* on the rate of diarrhea of weaned piglets are limited. The current study demonstrated that the dietary supplementation of *S. boulardii* mafic-1701 significantly decreased the rate of diarrhea over the entire 4 weeks.

It is generally known that weaning could lead to breakdown of intestinal barrier functions [24, 25]. When the intestinal barrier is damaged, microbial colonization increases the risk of inflammation [26]. In this study, we found that the levels of pro-inflammatory cytokines TNF-α and IL-6 were decreased in SB group compared with CON group, but there were no significant differences on the levels of IL-8 and IL-4 among three treatment groups. A previous study also reported that *S. boulardii* could reduce TNF-α and IL-6 levels in mice ulcerative colitis carcinogenesis model [27]. These results indicated that *S. boulardii* mafic-1701 has beneficial effects on intestinal health by decreasing inflammation. Previous studies showed that *S. boulardii* blocked nuclear factor kappa B activation and reduced colonic inflammation [28, 29]. Thus, we speculate that *S. boulardii* mafic-1701 altered the levels of pro-inflammatory cytokines through modulation of the signaling pathway involved in the inhibition of nuclear factor kappa B activated pathways. In addition, other previous studies have reported that mucus is composed of many immunomodulatory molecules with mucins forming the basic skeleton, which protect intestinal epithelial barrier integrity and reduce pro-inflammatory responses [4, 30]. Caballero-Franco et al. demonstrated that oral administration of probiotic increased mucin gene expression and secretion [31]. Therefore, it is speculated that *S. boulardii* mafic-1701 has a modulatory effect on inflammatory responses that correlates with the regulation of mucin transcription.

Probiotics can activate the local mucosal protective mechanisms and exert beneficial effects on the host such as modulate anti-oxidation and immune responses [32, 33]. In our study, we observed that *S. boulardii* mafic-1701 and aureomycin supplementation had no effect on IgA and IgG levels in the serum. In terms of antioxidant analysis, we found that T-SOD was increased in the SB group of the piglets, which suggests *S. boulardii* mafic-1701 plays a role in improving antioxidant capacity and protecting intestinal mucosa [33].

The diversity of the microorganisms in the mammalian gut is very large. It has been estimated that 500–1000 bacterial species inhabit the gut [34]. The gut microbiota has a symbiotic relationship with the host. Oral ingestion of a feed additive can regulate the delicate balance between host and microbes. From the results of phylum analysis, we found that the cecum microbial floras were dominated by Firmicutes, which is consistent with previous findings reported by Yu et al. [35]. Wang et al. reported a significant increase in abundance of Firmicutes and decrease in abundance of Bacteroidetes in which piglets were fed probiotics [14]. In the present study, compared to CON group, we found an increased abundance of Proteobacteria and decreased abundance of Firmicutes and Bacteroidetes in the colon in SB group. Difference between the Wang et al. study and this study may be attributed to the use of different probiotic strains. Indeed, different probiotic strains could exert different physiological effects.

From current study, *S. boulardii* mafic-1701 inclusion resulted in higher bacterial diversity in cecum and colon of piglets. The population of *Ruminococcaceae_UCG_099* and *Turicibacter* genus were significantly increased in cecum of SB group compared to CON group. These bacteria are believed to be significant producers of SCFAs, which are intestinal epithelial energy components that have anti-inflammatory properties and protect intestinal epithelial cells [36–38]. In addition, Ruminococcaceae can utilize diverse polysaccharides [39]. Indeed, the yeast cell wall consists of mannose, chitin, 1,3-β-glucan and 1,6-β-glucan [6]. Therefore, the increased population of *Ruminococcaceae_UCG_099* in the cecum might be due to *S. boulardii* mafic-1701 being used as a substrate source to stimulate proliferation of *Ruminococcaceae_UCG_099*. *S. boulardii* mafic-1701 inclusion showed some alterations with regard to microbiota communities. In the colon, *S. boulardii* mafic-1701 inclusion increased the abundance of *Bacillus* genus, which have excellent immunomodulatory and anti-inflammatory efficacy [40, 41]. In addition, a previous study reported that several *Bacillus* species, reduced pathogen colonization but the mechanisms by which this occurs is unclear [42]. Notably, the relative abundance of Clostridiaceae_4 family, which are negatively linked with antibiotic-associated diarrhea and colitis, was significantly increased in ANT group compared with SB group. It has been demonstrated that antibiotic treatment alters the composition of gut microbiota, manifesting the host susceptible to pathogen infection [24, 43].

Microbially-produced SCFAs as crucial in regulating health of the host and play a central role in gut metabolism [44]. A previously published report indicated that probiotics can increase SCFAs production [14]. In this study, *S. boulardii* mafic-1701 supplementation increased the concentrations of cecal isobutyrate and valerate. Compared with the other two groups, ANT group increased the concentrations of colonic propionate and butyrate. The increase of SCFAs production may be associated with the types of food where the fed intake is the most significant variable [45].

## Conclusion

In conclusion, dietary supplementation of *S. boulardii* mafic-1701 improved F:G, decreased the rate of diarrhea, increased the serum concentration of T-SOD, and decreased the levels of TNF-α, IL-6 in jejunum of weaned piglets. In addition, diets supplemented with *S. boulardii* mafic-1701 enriched the abundance of *Ruminococcaceae_UCG_099*, *Turicibacter* in the cecal digesta, which may contribute to the higher concentrations of cecal SCFAs. These findings suggest that supplementation of *S. boulardii* mafic-1701 in diets improved feed conversion ratio and antioxidant capacity, alleviated diarrhea and inflammation, and regulated gut health by promoting beneficial bacteria and their fermentation metabolites in weaned piglets.

## Data Availability

The data analyzed during the current study are available from the corresponding author on reasonable request.

## Abbreviations

*S. boulardii*: *Saccharomyces boulardii*
ADG: Average daily gain
ADFI: Average daily feed intake
F:G: Feed to gain ratio
SCFAs: Short chain fatty acids
T-SOD: Total superoxide dismutase
MDA: Malondialdehyde
T-AOC: Total antioxidant capacity
GSH-Px: Glutathione peroxidase
IL-8: Interleukin-8
IL-4: Interleukin-4
IL-6: Interleukin-6
TNF-α: Tumor necrosis factor-α
OTUs: Operational taxonomic units
PCA: Principal component analysis
LDA: Linear discriminant analysis
LEfSe: Linear discriminant analysis effect size
